# Intermittent Fasting: A User-Friendly Method for Type 2 Diabetes Mellitus

**DOI:** 10.7759/cureus.19348

**Published:** 2021-11-08

**Authors:** Mahreen Saeed, Moeez Ali, Tehreem Zehra, Saiyed Ali Haider Zaidi, Rihab Tariq

**Affiliations:** 1 Internal Medicine, Jamal Noor Hospital, Karachi, PAK; 2 Internal Medicine, California Institute of Behavioral Neurosciences and Psychology, Fairfield, USA; 3 Internal Medicine, Saifee Hospital, Karachi, PAK; 4 Anesthesiology, Sindh Institute of Urology and Transplantation, Karachi, PAK

**Keywords:** type 1 diabetes mellitus, type 2 diabetes mellitus, intermittent fasting, fasting, weight loss and obesity, glycated hemoglobin (hba1c), therapeutic fasting, religious fasting

## Abstract

Type 2 diabetes mellitus (T2DM) is an alarmingly rising disorder characterized mainly by insulin resistance and hyperglycemia. Due to the impairment of glucose homeostasis, most subjects present with elevated serum glucose levels, which can lead to several complications, including hospitalizations and even death. Diet quality and quantity are at the heart of its pathogenesis; hence, for the management of this condition, a technique known as intermittent fasting (IF) has been an area of interest for researchers. Different fasting regimens, including alternate-day fasting, religious fasting, and time-restricted fasting, have proven to be of strategic importance for glycemic control due to their physiologic effects. According to case studies and randomized trials, therapeutic fasting has been shown to reverse insulin resistance, resulting in the discontinuance of insulin therapy while maintaining blood sugar levels.

Studies on IF have demonstrated their efficacy in glycemic control and other metabolic parameters, including reducing visceral fat and controlling inflammatory mediators and markers such as C-reactive protein (CRP) and interleukin-6 (IL-6), but control in obesity is its most significant effect as it acts as a risk factor for T2DM. Several case studies have shown a reduction in elevated hemoglobin A1c (HbA1c) levels in subjects after fasting, which some believe is due to sirtuin-6 (SIRT6) proteins. SIRT6 proteins are believed to be responsible for blood glucose homeostasis and insulin resistance reversal by increasing its sensitivity. This family of proteins is increased after fasting; hence, further research in this area will help researchers better understand its mechanism of action and potential therapeutic effects on T2DM. With an alarming increase in the incidence of T2DM around the world, a cost-effective strategy is required to control the disease with easy patient compliance, and IF might prove to be the solution.

## Introduction and background

Intermittent fasting (IF), also known as periodic fasting or intermittent energy reduction, is proportionally a new dietary proposal to weight regulation and includes inventive regular caloric intake within a small and specific period [1]. Intermittent fasting has achieved a lot of regards in terms of weight reduction. IF is of several categories and mainly cycles between periods of caloric restriction and eating [2]. Different patterns of intermittent fasting have been proposed, the most popular of which is the alternate-day fasting, which usually involves a fasting day followed by a feeding day. These patterns could be followed for days to weeks to months in order to regulate weight and maintain blood glucose levels [3].

Time-restricted fasting is also one of the patterns of intermittent fasting, which usually involves food intake in a particular period (3-12 hours) and fasting for the remaining hours of the day. Ramadan fasting is an excellent illustration of time-restricted fasting. During the holy month of Ramadan, Muslims from all over the world fast during daylight, followed by the consumption of food and drinks after sunset until the first light of the sun [4].

Diabetes mellitus is a long-standing metabolic disease, which signalizes raised sugar levels in the blood. The disease progressively leads to damage to various other organs such as the heart, kidney, eyes, nerves, and other vessels. According to the World Health Organization (WHO), over 90% of cases are type 2 diabetes mellitus (T2DM), a condition that is marked by decreased production of insulin by beta cells of the pancreas and variable magnitude of insulin resistance [5,6]. The incidence of this disease is multiplying worldwide predominantly due to environmental, genetic, and metabolic factors [7-11]. The risk factor for T2DM could be categorized into modifiable risk factors, such as decreased physical activity, raised BMI, and unhealthy diet, and non-modifiable risk factors, such as age, ethnicity, and positive family history [12,13].

The objective of this article is to highlight the use of IF for the management of T2DM. Substantial data is available on this subject in the form of case studies and other types of literature mentioned in the subsequent parts of the article. This article will feature the types of IF, their physiological effects on diabetics, and studies involving human subjects for T2DM management strategy.

## Review

Adaptive responses of humans to intermittent fasting

Fasting is deliberate self-abnegation from food, and intermittent fasting (IF) is a methodology in which an eating regimen proposes consumption of food only within a specified time of the day, succeeded by fasting [14]. All formats of IF mainly restrict carbohydrate intake, which results in reduced energy requirement by the body and increases hunger. This primarily occurs due to compensatory hormonal changes regulating body weight [15]. As defined by the American Diabetes Association (ADA) and the European Association for the Study of Diabetes, a reduction in carbohydrate intake is considered to be a useful treatment of type 2 diabetes (T2D) in standard medical nutrition therapy [16]. The aforementioned approach precedes even the development of exogenous insulin in 1921 as a novel treatment modality. It is premised on the evidence that carbohydrates are the macronutrient with the highest insulin and macronutrient indices [17].

In humans, glucose is primarily stored in the liver in the form of glycogen. Depending on the physical and exertion activity, there is a 20% or greater reduction in serum glucose levels during fasting. Consequently, non-hepatic glucose, fat-derived ketone bodies, and free fatty acids are utilized as an alternative energy source [18]. Although most tissues can metabolize fatty acids for energy, during persistently long durations of fasting, the brain uses β-hydroxybutyrate and acetoacetate in addition to glucose. It is interesting to note that after hepatic exhaustion of glycogen, approximately 80 g/day of glucose is generated by gluconeogenesis. According to a study, the ketone bodies, free fatty acids, and gluconeogenesis allow the majority of human beings to survive 30 or more days without food [19]. The degree to which fasting affects human metabolism and health outcomes depends on the type of fasting, which is mentioned in Table 1 [20].

**Table 1:** Types of fasting and their structure

Type	Fasting regimen
Alternate-day fasting regimen	Comprises alternate eating days (ad libitum consumption of food and beverages) with alternating fasting days (abstaining from energy-providing food and beverages)
Time-restricted food consumption	Ad libitum food intake at different intervals in a particular time frame
Modified fasting regimen	Permits ad libitum eating five days of the week and restricting food in the remaining two to only 20%–25% of energy requirement
Religious fasting	A fast practiced by Muslims in their holy month of Ramadan starting a little before sunrise to sunset. Eating is permitted before their morning prayer time and after the sunset. Thus, the average Ramadan fasting time around the world is about 12 hours
Other religious fasting	Fasting practices for religious or spiritual purposes

In China, a prospective, single-blind randomized controlled trial (RCT) was performed between December 2015 and December 2016 [21]. The subjects were randomly allocated to receive either a low carbohydrate diet (LCD) or a low-fat diet (LFD). Before the intervention, all subjects underwent a one-week washout period to diminish the effect of background diets on the study [22]. The effect of restricting the diet was remarkable. Compared with the baseline, hemoglobin A1c (HbA1c) levels in both the LCD group and the LFD group decreased significantly (0.63% ± 1.18% and 0.31% ± 0.70%, respectively). Furthermore, when compared with the baseline, the dosage of insulin used in the two groups also decreased significantly after the intervention (p < 0.05) [21].

In addition to this, another randomized non-inferiority trial was conducted between April 7, 2015, and September 7, 2017, at the University of South Australia [23]. In this study, 137 adults (n = 137) with type 2 diabetes were randomized 1:1 to parallel diet groups (intermittent energy restriction [n = 70] or continuous energy restriction [n = 67]). The intervention mainly restricted diet (500-600 kcal/day) followed for two nonconsecutive days per week (participants followed their usual diet for the other five days) or a continuous energy restriction diet (1200-1500 kcal/day) followed for seven days per week for 12 months [23]. The notable outcome was the change in hemoglobin A1c (HbA1c) level, with equivalence prespecified by a 90% confidence interval (CI) margin of ±0.5%. The secondary outcome was weight reduction with equivalence set at ±2.5 kg (±1.75 kg for fat mass loss and ±0.75 kg for fat-free mass loss) [23]. This data proves that dietary intervention is a strategy to manage T2DM, as it can reduce the burden on islet cells and thus improve blood glucose levels, lipid profiles, and cognitive status [24-26].

In addition to this, an animal study provided mechanistic clues for a better understanding of the effects of IF on glycemic control. It suggested that IF may slow or even reverse the development of type 2 diabetes (T2D) [27]. Mice were controlled with a fasting-mimicking diet (alternate-day fasting), which presented with a rise in the number of insulin-generating pancreatic beta cells. Differentiated cells in the pancreas decreased initially in the fasting state, and interestingly, the pancreatic transitional cells and beta cells increased in the non-fasting state after the initial fasting. This proves that in animal studies, intermittent energy restriction appears to be equally effective as, if not more effective than, continuous energy restriction for the reduction of disease risk factors [27].

Religious fasting

For physical and spiritual benefits, fasting is an essential part of several religions and cultures around the world. For example, fasting for Muslims in the holy month of Ramadan is an important religious practice. Adult Muslims fast from a little before sunrise until sunset, abstaining from any type of food or fluid intake, including oral medications and cigarette smoking [20]. According to a 2012 meta-analysis of 35 studies, weight changes were seen in subjects fasting during Ramadan. The subjects aged between 18 and 58 presented with a 1.24 kg reduction during this practice; this trend was noticed in 21 (60%) studies [28]. Another meta-analysis of 30 cohort studies investigated the changes in physiological biomarkers in fasting men and women in addition to weight loss [29]. The results showed that fasting blood glucose and low-density lipid (LDL) levels in both genders were reduced after Ramadan as compared with before it. It is interesting to note that the males experienced a significant reduction in their weight, triglycerides (TG), and total cholesterol levels and that the females experienced a rise in their high-density lipid (HDL) cholesterol levels [10]. Moreover, studies have also reported attenuated concentrations of pro-inflammatory markers such as C-reactive protein (CRP), interleukin-6 (IL-6), and tumor necrosis factor-alpha (TNF-α) associated with Ramadan fasting [30,31]. In addition, glycemic control is also improved during Ramadan fasting, and recent studies provided evidence suggesting that there are a 0.5 point decrease in hemoglobin A1c (HbA1c) levels in patients with type 2 diabetes after fasting during Ramadan [32].

In addition to Ramadan fasting, other religious fasts are also practiced globally. For example, a study involving 448 patients from hospitals in Utah found that the followers of The Church of Jesus Christ of Latter-day Saints who routinely fasted demonstrated lower fasting glucose levels, lower weight, and lower diabetes prevalence with an odds ratio (OR) of 0.41 and 95% confidence interval (CI) of 0.17-0.99 [33]. Substantial observational data is available on different types of fasting regimens, including religious fasting; however, participation of these individuals in clinical trials and studies remains low due to their reservations. It is suggested that a better reach-out program to these individuals is needed and that more epidemiological studies can improve our insight on the different types of fasting. Also, dietary nutritionists should be involved in these studies to identify the dietary changes adopted by the subjects and how they vary among individuals.

Therapeutic fasting, obesity, and type 2 diabetes mellitus

The symptoms of diabetes can be managed using medications; however, medications cannot completely cure the underlying condition [34]. As noted in an open-label Diabetes Remission Clinical Trial (DiRECT), caloric limitation and weight management are important factors for T2D. The DiRECT study demonstrated remission and maintenance of T2D via caloric restriction (~840 calories/day) and weight loss in a non-insulin-dependent diabetic population [35]. According to a case report, in June 2016, at age 54 years, a woman with a 15-year history of T2D and using metformin 1000 mg twice a day presented with elevated HbA1c levels (8.7%) [36]. The subject initiated a ketogenic diet (KD) and IF in July 2018 when her HbA1c was 9.3%. Interestingly, after a few months (in March 2019), her HbA1c, weight, and BMI changed from 9.3% to 6.4%, 55.3 to 54.9 kg, and 22.3 to 22.1 kg/m^2^, respectively [36]. Four weeks after her dietary changes, the patient reportedly discontinued all her medications, including metformin, a statin, and an antihypertensive drug, and at the same time, she improved her glycemic control [36]. On follow-up, the patient expressed her plans to maintain her KD and IF, which she currently continues. This is one of the strong evidence on how dietary changes, especially IF, can help in glycemic control in diabetic patients as the patient was seen to improve her glycemic control without using most of her medicine as mentioned above and was able to significantly reduce her HbA1c levels by fasting.

Another interesting case series involving three patients with T2D discusses the physiological changes seen after adopting dietary changes. Patient details and characteristics are summarized in Table 2 [37].

**Table 2:** Patient details and characteristics T2D: type 2 diabetes; HTN: hypertension; CKD: chronic kidney disease; RCC: renal cell carcinoma

	Sex	Age	Onset of T2D	History/comorbidities	Fasting regimen
Patient 1	Male	40	Present; 20 years prior	HTN, hypercholesterolemia	Three times/week for seven months
Patient 2	Male	52	Present; 25 years prior	CKD, RCC (nephrectomy in 2004), HTN, hypercholesterolemia	Three times/week for 11 months
Patient 3	Male	67	Present; 10 years prior	HTN, hypercholesterolemia	Alternating days for 11 months

Several changes were noted after the initiation of IF in all three subjects, which are summarized in Table 3 [36].

**Table 3:** Glycemic and other changes noted on patient follow-up IU: international units

	Initial HbA1c (%, mmol/mol)	Final HbA1c (%, mmol/mol)	Initial weight (kg)	Final weight (kg)	Initial diabetic medications	Final diabetic medications	Time to discontinuation of insulin (days)
Patient 1	11, 96.7	7, 53	83.8	73.8	Insulin glargine 58, insulin aspart 22, canagliflozin 300 mg, metformin 1 g	Canagliflozin 300 mg	5
Patient 2	7.2, 55.2	6, 42.1	61	50.4	Insulin lispro mix 25–38/32 IU 25	None	18
Patient 3	6.8, 50.8	6.2, 44.3	97.1	88.1	Metformin 1000 mg, insulin lispro mix 25–30/20 IU	None

The subjects were only allowed to consume dinner on fasting days; however, low carbohydrate lunch and dinner were consumed on non-fasting days. In addition to this, the patients were examined twice a month, and their laboratory results were recorded [37]. The most remarkable development of this case series was noted to be the discontinuation of insulin in all three patients, which strengthens the evidence for IF as an effective management modality for patients with T2D [37].

Another ongoing research in this area involves sirtuin proteins, particularly sirtuin-6 (SIRT6), which is believed to have a potential therapeutic effect on insulin resistance [19]. According to animal studies, it does this by enhancing insulin sensitivity and decreasing fasting blood glucose levels [38-40]. Both short-term and long-term caloric restriction has presented with elevated SIRT6 levels in animal data, which draws the researcher’s attention toward the role of IF in disease modification [41].

Although all patients suffering from T2D are not overweight, IF regimens mainly focus on bodyweight reduction as obesity is a known precipitating factor for T2D. Bariatric surgery is an effective strategy to manage obesity; however, it is limited by its accessibility, potential for complications, and invasive nature [15]. Although modifications in one’s lifestyle are highly recommended to manage T2D, attaining the desired glycemic control seems very complicated in obese patients, and this leads to the recommendation of therapeutic fasting. This restricts the caloric intake in obese patients and can help manage T2D without any invasive technique such as Bariatric surgery.

## Conclusions

The objective of this review article is to shed light on intermittent fasting as a lifestyle modification in patients with T2D and its outcomes. The effects of intermittent fasting on diabetics deserve more than a casual mention as it precipitates notable physiologic changes helping in the condition. Different studies carried out on people practicing religious fasting presented a noteworthy reduction in their weight in addition to reduced LDL and TG levels after fasting. Decreased HbA1c and lowered pro-inflammatory mediators such as TNF-α and IL-6 were also seen. As apparent from the mentioned studies, the patient compliance for this approach was very high in people who already practiced religious fasting. Also, in patients with complete novelty to this methodology, the feedback was very positive, and several patients remarked on enjoying being actively involved in the process of managing their diabetes.

Dietary modifications such as LCD and LFD have also been found to be effective in the treatment of obesity, and apart from significantly reducing weight, it can also effectively improve blood lipid and insulin resistance. Educating patients on the advantages of fasting in the management of T2D may aid in the remission of the disease and curtail the use of pharmacological interventions. Further studies and more clinical trials will help researchers understand the exact mechanism of IF on diabetics, and there is substantial data available to encourage research in this area. Furthermore, if validated, this approach will revolutionize our understanding and management strategy toward this rising and chronic disease.
